# Bis[2,6-bis­(1*H*-benzimidazol-2-yl)pyridine]­nickel(II) dipicrate dimethyl­formamide disolvate

**DOI:** 10.1107/S160053681002773X

**Published:** 2010-07-17

**Authors:** Xingcai Huang, Fan Kou, Baoliang Qi, Xuan Meng, Huilu Wu

**Affiliations:** a School of Chemical and Biological Engineering, Lanzhou Jiaotong University, Lanzhou 730070, People’s Republic of China

## Abstract

In the title compound, [Ni(C_19_H_13_N_5_)_2_](C_6_H_2_N_3_O_7_)_2_·2C_3_H_7_NO, the Ni^II^ ion is coordinated by two tridentate 2,6-bis­(1*H*-benzimidazol-2-yl)pyridine ligands in a distorted octa­hedral geometry. In the crystal structure, the picrate anions and solvent dimethyl­formamide (DMF) mol­ecules are connected to the cation *via* inter­molecular N—H⋯O hydrogen bonds. Further stabilization is provided by weak inter­molecular C—H⋯O hydrogen bonds. One of the DMF moleclues is disordered over two sites with refined occupancies of 0.737 (3) and 0.263 (3).

## Related literature

For a related structure, see: Freire *et al.* (2003 ▶).
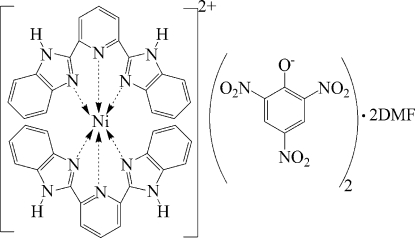


## Experimental

### 

#### Crystal data

[Ni(C_19_H_13_N_5_)_2_](C_6_H_2_N_3_O_7_)_2_·2C_3_H_7_NO
*M* *_r_* = 1283.80
Monoclinic, 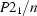

*a* = 14.2087 (3) Å
*b* = 26.5215 (5) Å
*c* = 14.6989 (3) Å
β = 93.775 (1)°
*V* = 5527.06 (19) Å^3^
*Z* = 4
Mo *K*α radiation
μ = 0.44 mm^−1^
*T* = 153 K
0.36 × 0.25 × 0.19 mm

#### Data collection

Rigaku R-AXIS Spider diffractometer
Absorption correction: multi-scan (*ABSCOR*; Higashi, 1995 ▶) *T*_min_ = 0.857, *T*_max_ = 0.921
52251 measured reflections
12544 independent reflections
9403 reflections with *I* > 2σ(*I*)
*R*_int_ = 0.038

#### Refinement

*R*[*F* ^2^ > 2σ(*F* ^2^)] = 0.040
*wR*(*F* ^2^) = 0.106
*S* = 1.11
12544 reflections
814 parameters
16 restraints
H-atom parameters constrained
Δρ_max_ = 0.72 e Å^−3^
Δρ_min_ = −0.60 e Å^−3^

### 

Data collection: *RAPID-AUTO* (Rigaku/MSC (2004 ▶); cell refinement: *RAPID-AUTO*; data reduction: *RAPID-AUTO*; program(s) used to solve structure: *SHELXS97* (Sheldrick, 2008 ▶); program(s) used to refine structure: *SHELXL97* (Sheldrick, 2008 ▶); molecular graphics: *PLATON* (Spek, 2009 ▶); software used to prepare material for publication: *SHELXTL* (Sheldrick, 2008 ▶).

## Supplementary Material

Crystal structure: contains datablocks global, I. DOI: 10.1107/S160053681002773X/lh5079sup1.cif

Structure factors: contains datablocks I. DOI: 10.1107/S160053681002773X/lh5079Isup2.hkl

Additional supplementary materials:  crystallographic information; 3D view; checkCIF report

## Figures and Tables

**Table 1:** Hydrogen-bond geometry (Å, °)

*D*—H⋯*A*	*D*—H	H⋯*A*	*D*⋯*A*	*D*—H⋯*A*
N7—H7*A*⋯O8	0.88	1.93	2.769 (2)	158
C24—H24*A*⋯O14	0.95	2.60	3.226 (3)	124
C28—H28*A*⋯O8	0.95	2.26	3.144 (2)	155
C51—H51*A*⋯O15	0.98	2.41	2.812 (4)	104
C51—H51*C*⋯O7	0.98	2.47	3.304 (4)	143
C54—H54*A*⋯O16	0.98	2.43	2.800 (3)	102
N2—H2*B*⋯O16^i^	0.88	1.91	2.777 (2)	170
C9—H9*A*⋯O16^i^	0.95	2.55	3.411 (3)	150
N5—H5*B*⋯O1^ii^	0.88	1.81	2.684 (2)	175
N10—H10*B*⋯O15^iii^	0.88	1.92	2.803 (3)	180
C10—H10*A*⋯O6^iv^	0.95	2.59	3.398 (3)	143
C55—H55*A*⋯O10^v^	0.98	2.49	3.326 (3)	143

Symmetry codes: (i) 
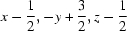
; (ii) 
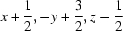
; (iii) 
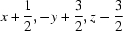
; (iv) 
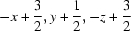
; (v) 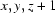.

**Fig. 1.**
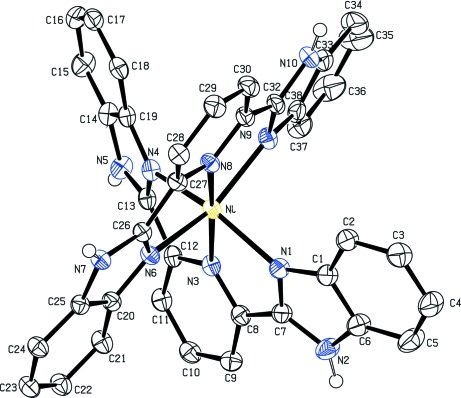
The cation of the title compound with displacement ellipsoids drawn at the 30% probability level. H atoms bonded to C atoms have been omitted for clarity.

**Fig. 2.**
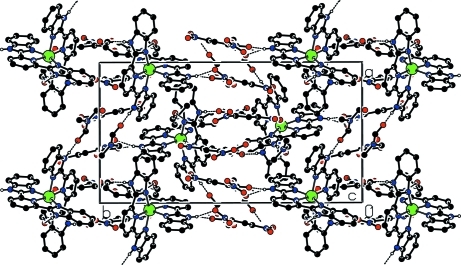
Part of the crystal structure of the title compound with hydrogen bonds drawn as dashed lines. The disorder is not shown.

## supplementary crystallographic information

### Comment

The asymmetric unit of the title complex (Fig. 1) consists of a [Ni^II^(bbp)_2_] cations (bbp = 2,6-bis(1*H*-benzimidazol-2-yl)pyridine) two picrate anions, and two DMF solvate molecules. The Ni^II^ ion is coordinated by two tridentate bbp ligands in a distorted octahedral geometry. The Ni-N bond distances are comparable to those in a related structure Freire *et al.* (2003).

In the crystal structure, the picrate anions and solvent dimethylformamide (DMF) molecules are connected to the cation via intermolecular N-H···O hydrogen bonds (Fig. 2). One of the DMF moleclues is disordered over two sites with refined occupancies  of 0.737 (3) and 0.263 (3).

### Experimental

To a stirred solution of 2,6-bis(2-benzimidazolyl)pyridine (0.1557 g, 0.50 mmol) in hot MeOH (10 ml), Ni(picrate)_2_ (0.1287 g, 0.25 mmol) solution dissolved in MeOH (5 ml) was added. Owing to the formation of [Ni^II^(bbp)_2_] complex, the pale yellow precipitate was generated immediately. The sediment was filtered, washed with MeOH and absolute Et_2_O, and dried *in vacuo*. The dried precipitate was dissolved in DMF to form a yellow solution that was allowed to evaporate at room temperature. The dried precipitate was dissolved in DMF and yellow crystals suitable for X-ray diffraction studies were obtained by ether diffusion into this solution after several days at room temperature (found: C, 52.25; H, 2.74; N, 20.15. Calcd. for C_56_ H_44_ N_18_ O_16_ Ni: C, 52.79; H, 2.64; N, 19.70).

### Refinement

All H atoms were found in difference Fourier maps and were subsequently refined in a riding-model approximation with C—H = 0.95-0.98; N-H = 0.88Å and *U*_iso_(H) = 1.2 *U*_eq_(C,N) or 1.5 *U*_eq_(C_methyl_).

### Crystal data

**Table:**

[Ni(C_19_H_13_N_5_)_2_](C_6_H_2_N_3_O_7_)_2_·2C_3_H_7_NO	*F*(000) = 2648
*M**_r_* = 1283.80	*D*_x_ = 1.543 Mg m^−^^3^
Monoclinic, *P*2_1_/*n*	Mo *K*α radiation, λ = 0.71073 Å
Hall symbol: -P 2yn	Cell parameters from 12544 reflections
*a* = 14.2087 (3) Å	θ = 3.0–27.5°
*b* = 26.5215 (5) Å	µ = 0.44 mm^−^^1^
*c* = 14.6989 (3) Å	*T* = 153 K
β = 93.775 (1)°	Block, yellow
*V* = 5527.06 (19) Å^3^	0.36 × 0.25 × 0.19 mm
*Z* = 4	

### Data collection

**Table:**

Rigaku R-AXIS Spider diffractometer	12544 independent reflections
Radiation source: fine-focus sealed tube	9403 reflections with *I* > 2σ(*I*)
graphite	*R*_int_ = 0.038
ω scans	θ_max_ = 27.5°, θ_min_ = 3.0°
Absorption correction: multi-scan (*ABSCOR*; Higashi, 1995)	*h* = −18→18
*T*_min_ = 0.857, *T*_max_ = 0.921	*k* = −34→32
52251 measured reflections	*l* = −18→19

### Refinement

**Table:**

Refinement on *F*^2^	Primary atom site location: structure-invariant direct methods
Least-squares matrix: full	Secondary atom site location: difference Fourier map
*R*[*F*^2^ > 2σ(*F*^2^)] = 0.040	Hydrogen site location: inferred from neighbouring sites
*wR*(*F*^2^) = 0.106	H-atom parameters constrained
*S* = 1.11	*w* = 1/[σ^2^(*F*_o_^2^) + (0.0471*P*)^2^ + 2.435*P*] where *P* = (*F*_o_^2^ + 2*F*_c_^2^)/3
12544 reflections	(Δ/σ)_max_ = 0.002
814 parameters	Δρ_max_ = 0.72 e Å^−^^3^
16 restraints	Δρ_min_ = −0.60 e Å^−^^3^

### Special details

**Table:**

Geometry. All e.s.d.'s (except the e.s.d. in the dihedral angle between two l.s. planes) are estimated using the full covariance matrix. The cell e.s.d.'s are taken into account individually in the estimation of e.s.d.'s in distances, angles and torsion angles; correlations between e.s.d.'s in cell parameters are only used when they are defined by crystal symmetry. An approximate (isotropic) treatment of cell e.s.d.'s is used for estimating e.s.d.'s involving l.s. planes.
Refinement. Refinement of *F*^2^ against ALL reflections. The weighted *R*-factor *wR* and goodness of fit *S* are based on *F*^2^, conventional *R*-factors *R* are based on *F*, with *F* set to zero for negative *F*^2^. The threshold expression of *F*^2^ > σ(*F*^2^) is used only for calculating *R*-factors(gt) *etc*. and is not relevant to the choice of reflections for refinement. *R*-factors based on *F*^2^ are statistically about twice as large as those based on *F*, and *R*- factors based on ALL data will be even larger.

### Fractional atomic coordinates and isotropic or equivalent isotropic displacement parameters (Å^2^)

**Table:**

	*x*	*y*	*z*	*U*_iso_*/*U*_eq_	Occ. (<1)
Ni	0.952407 (18)	0.808293 (9)	0.021698 (16)	0.01830 (7)	
N1	0.81300 (12)	0.82406 (6)	−0.02938 (11)	0.0216 (3)	
N2	0.68026 (13)	0.86678 (7)	−0.00986 (12)	0.0297 (4)	
H2B	0.6386	0.8853	0.0167	0.036*	
N3	0.90743 (12)	0.86064 (6)	0.11151 (10)	0.0198 (3)	
N4	1.07615 (12)	0.82338 (6)	0.10559 (10)	0.0196 (3)	
N5	1.14391 (12)	0.87962 (6)	0.20267 (11)	0.0237 (4)	
H5B	1.1495	0.9036	0.2440	0.028*	
N6	0.91013 (12)	0.74464 (6)	0.09522 (10)	0.0192 (3)	
N7	0.90002 (12)	0.66063 (6)	0.10016 (10)	0.0196 (3)	
H7A	0.9042	0.6289	0.0833	0.024*	
N8	0.98180 (12)	0.74986 (6)	−0.06205 (10)	0.0192 (3)	
N9	1.01771 (12)	0.84446 (6)	−0.08505 (11)	0.0222 (4)	
N10	1.08396 (13)	0.83546 (7)	−0.21762 (11)	0.0266 (4)	
H10B	1.1024	0.8212	−0.2675	0.032*	
C1	0.75258 (15)	0.81827 (8)	−0.10674 (13)	0.0235 (4)	
C2	0.76573 (15)	0.79269 (8)	−0.18759 (14)	0.0266 (4)	
H2A	0.8209	0.7733	−0.1949	0.032*	
C3	0.69571 (17)	0.79652 (9)	−0.25670 (14)	0.0323 (5)	
H3A	0.7030	0.7796	−0.3128	0.039*	
C4	0.61400 (17)	0.82489 (10)	−0.24579 (15)	0.0377 (6)	
H4A	0.5676	0.8271	−0.2952	0.045*	
C5	0.59884 (17)	0.84959 (10)	−0.16568 (16)	0.0393 (6)	
H5A	0.5429	0.8684	−0.1583	0.047*	
C6	0.66955 (16)	0.84560 (9)	−0.09641 (14)	0.0286 (5)	
C7	0.76675 (14)	0.85343 (8)	0.02569 (13)	0.0225 (4)	
C8	0.81559 (15)	0.87180 (7)	0.11020 (13)	0.0217 (4)	
C9	0.77778 (15)	0.89747 (8)	0.18153 (13)	0.0241 (4)	
H9A	0.7123	0.9048	0.1808	0.029*	
C10	0.83956 (15)	0.91218 (7)	0.25447 (13)	0.0242 (4)	
H10A	0.8155	0.9290	0.3051	0.029*	
C11	0.93499 (15)	0.90269 (7)	0.25414 (13)	0.0233 (4)	
H11A	0.9775	0.9139	0.3025	0.028*	
C12	0.96725 (14)	0.87593 (7)	0.18021 (12)	0.0200 (4)	
C13	1.06310 (14)	0.85976 (7)	0.16579 (12)	0.0200 (4)	
C14	1.21635 (15)	0.85501 (7)	0.16295 (13)	0.0228 (4)	
C15	1.31347 (16)	0.86088 (8)	0.17293 (14)	0.0294 (5)	
H15A	1.3423	0.8853	0.2131	0.035*	
C16	1.36581 (16)	0.82949 (9)	0.12155 (14)	0.0293 (5)	
H16A	1.4326	0.8325	0.1261	0.035*	
C17	1.32360 (16)	0.79310 (8)	0.06248 (13)	0.0260 (4)	
H17A	1.3626	0.7718	0.0292	0.031*	
C18	1.22709 (15)	0.78752 (7)	0.05159 (13)	0.0229 (4)	
H18A	1.1988	0.7630	0.0114	0.027*	
C19	1.17274 (14)	0.81968 (7)	0.10238 (12)	0.0202 (4)	
C20	0.87517 (14)	0.73085 (7)	0.17740 (12)	0.0202 (4)	
C21	0.84671 (15)	0.76066 (8)	0.24916 (13)	0.0242 (4)	
H21A	0.8505	0.7964	0.2470	0.029*	
C22	0.81314 (16)	0.73620 (8)	0.32282 (13)	0.0258 (4)	
H22A	0.7928	0.7555	0.3723	0.031*	
C23	0.80823 (15)	0.68362 (8)	0.32654 (13)	0.0258 (4)	
H23A	0.7850	0.6681	0.3788	0.031*	
C24	0.83610 (15)	0.65352 (8)	0.25659 (13)	0.0238 (4)	
H24A	0.8330	0.6178	0.2596	0.029*	
C25	0.86886 (14)	0.67821 (7)	0.18163 (12)	0.0195 (4)	
C26	0.92275 (14)	0.70159 (7)	0.05158 (12)	0.0190 (4)	
C27	0.95905 (14)	0.70291 (7)	−0.03946 (12)	0.0198 (4)	
C28	0.97005 (15)	0.66250 (7)	−0.09852 (13)	0.0223 (4)	
H28A	0.9525	0.6292	−0.0826	0.027*	
C29	1.00753 (15)	0.67264 (8)	−0.18119 (13)	0.0256 (4)	
H29A	1.0166	0.6458	−0.2225	0.031*	
C30	1.03200 (15)	0.72131 (8)	−0.20469 (13)	0.0258 (4)	
H30A	1.0579	0.7283	−0.2613	0.031*	
C31	1.01730 (14)	0.75933 (7)	−0.14277 (12)	0.0212 (4)	
C32	1.03792 (14)	0.81305 (8)	−0.15124 (12)	0.0222 (4)	
C33	1.09654 (15)	0.88502 (8)	−0.19215 (15)	0.0278 (5)	
C34	1.14196 (19)	0.92483 (9)	−0.23244 (19)	0.0431 (6)	
H34A	1.1675	0.9217	−0.2903	0.052*	
C35	1.1477 (2)	0.96901 (9)	−0.1837 (2)	0.0539 (8)	
H35A	1.1797	0.9968	−0.2081	0.065*	
C36	1.10840 (19)	0.97476 (9)	−0.0996 (2)	0.0457 (7)	
H36A	1.1147	1.0061	−0.0685	0.055*	
C37	1.06076 (16)	0.93587 (8)	−0.06091 (16)	0.0321 (5)	
H37A	1.0328	0.9399	−0.0044	0.038*	
C38	1.05538 (15)	0.89021 (8)	−0.10836 (14)	0.0253 (4)	
O1	0.65289 (12)	0.54468 (5)	0.82415 (10)	0.0306 (3)	
O2	0.62027 (14)	0.51404 (6)	0.64861 (10)	0.0410 (4)	
O3	0.67343 (13)	0.43908 (6)	0.62613 (10)	0.0400 (4)	
O4	0.59111 (12)	0.31139 (6)	0.84018 (11)	0.0351 (4)	
O5	0.59714 (13)	0.33068 (6)	0.98397 (11)	0.0403 (4)	
O6	0.64921 (15)	0.50176 (7)	1.07987 (11)	0.0502 (5)	
O7	0.58363 (14)	0.55750 (6)	0.98960 (12)	0.0450 (4)	
N11	0.64320 (14)	0.47168 (7)	0.67589 (12)	0.0294 (4)	
N12	0.59939 (13)	0.34216 (7)	0.90252 (13)	0.0292 (4)	
N13	0.61788 (13)	0.51554 (7)	1.00389 (12)	0.0306 (4)	
C39	0.63458 (15)	0.49941 (8)	0.83836 (14)	0.0240 (4)	
C40	0.63181 (15)	0.45938 (8)	0.77095 (13)	0.0241 (4)	
C41	0.62234 (15)	0.40924 (8)	0.79094 (14)	0.0251 (4)	
H41A	0.6232	0.3846	0.7441	0.030*	
C42	0.61151 (15)	0.39492 (8)	0.88062 (14)	0.0251 (4)	
C43	0.61092 (15)	0.43016 (8)	0.94946 (14)	0.0259 (4)	
H43A	0.6043	0.4199	1.0106	0.031*	
C44	0.62006 (14)	0.48017 (8)	0.92860 (13)	0.0240 (4)	
O8	0.88901 (12)	0.57028 (5)	0.00568 (9)	0.0300 (3)	
O9	0.92480 (11)	0.55055 (5)	−0.16937 (10)	0.0294 (3)	
O10	0.84170 (12)	0.48845 (6)	−0.22604 (10)	0.0347 (4)	
O11	0.82471 (14)	0.33880 (6)	−0.04445 (12)	0.0456 (5)	
O12	0.79563 (12)	0.34836 (6)	0.09758 (11)	0.0395 (4)	
O13	0.86025 (13)	0.50378 (7)	0.24763 (10)	0.0408 (4)	
O14	0.94923 (12)	0.55764 (6)	0.18430 (10)	0.0336 (4)	
N14	0.87920 (12)	0.51166 (6)	−0.16065 (11)	0.0234 (4)	
N15	0.81975 (14)	0.36483 (7)	0.02401 (13)	0.0314 (4)	
N16	0.89668 (13)	0.52102 (7)	0.18029 (11)	0.0259 (4)	
C45	0.88191 (14)	0.52360 (7)	0.00939 (13)	0.0210 (4)	
C46	0.87130 (14)	0.49064 (7)	−0.06978 (13)	0.0207 (4)	
C47	0.85235 (14)	0.44015 (8)	−0.06581 (14)	0.0231 (4)	
H47A	0.8454	0.4207	−0.1201	0.028*	
C48	0.84342 (15)	0.41773 (7)	0.01854 (14)	0.0244 (4)	
C49	0.85692 (14)	0.44525 (8)	0.09820 (14)	0.0234 (4)	
H49A	0.8515	0.4294	0.1556	0.028*	
C50	0.87808 (14)	0.49524 (7)	0.09376 (13)	0.0211 (4)	
O16	1.05337 (12)	0.58190 (7)	0.59231 (12)	0.0446 (4)	
N18	0.91649 (14)	0.54522 (7)	0.53721 (12)	0.0309 (4)	
C54	0.9070 (3)	0.57566 (11)	0.45561 (18)	0.0589 (9)	
H54A	0.9470	0.6056	0.4635	0.088*	
H54B	0.8411	0.5860	0.4443	0.088*	
H54C	0.9265	0.5560	0.4037	0.088*	
C55	0.8405 (2)	0.50987 (11)	0.55095 (17)	0.0467 (7)	
H55A	0.8561	0.4899	0.6060	0.070*	
H55B	0.8322	0.4874	0.4981	0.070*	
H55C	0.7819	0.5285	0.5580	0.070*	
C56	0.98743 (17)	0.55163 (9)	0.59809 (15)	0.0341 (5)	
H56	0.9883	0.5313	0.6513	0.041*	
O15	0.6423 (2)	0.71003 (13)	1.12342 (16)	0.0494 (4)	0.737 (3)
N17	0.6175 (3)	0.70137 (18)	0.97022 (17)	0.0494 (4)	0.737 (3)
C51	0.6925 (2)	0.66572 (14)	0.9597 (2)	0.0494 (4)	0.737 (3)
H51A	0.7282	0.6610	1.0184	0.074*	0.737 (3)
H51B	0.7346	0.6785	0.9149	0.074*	0.737 (3)
H51C	0.6657	0.6334	0.9387	0.074*	0.737 (3)
C52	0.5575 (3)	0.71317 (16)	0.8899 (2)	0.0494 (4)	0.737 (3)
H52A	0.5101	0.7380	0.9054	0.074*	0.737 (3)
H52B	0.5259	0.6824	0.8669	0.074*	0.737 (3)
H52C	0.5958	0.7271	0.8428	0.074*	0.737 (3)
C53	0.5989 (2)	0.71974 (15)	1.0503 (2)	0.0494 (4)	0.737 (3)
H53	0.5473	0.7425	1.0518	0.059*	0.737 (3)
O15'	0.6142 (6)	0.7010 (4)	1.1271 (4)	0.0494 (4)	0.263 (3)
N17'	0.6367 (8)	0.6933 (4)	0.9769 (3)	0.0494 (4)	0.263 (3)
C51'	0.6808 (7)	0.6646 (4)	0.9079 (5)	0.0494 (4)	0.263 (3)
H51D	0.7196	0.6378	0.9371	0.074*	0.263 (3)
H51E	0.7208	0.6869	0.8738	0.074*	0.263 (3)
H51F	0.6321	0.6496	0.8661	0.074*	0.263 (3)
C52'	0.5766 (6)	0.7345 (3)	0.9489 (5)	0.0494 (4)	0.263 (3)
H52D	0.5508	0.7499	1.0026	0.074*	0.263 (3)
H52E	0.5248	0.7222	0.9075	0.074*	0.263 (3)
H52F	0.6130	0.7598	0.9174	0.074*	0.263 (3)
C53'	0.6509 (6)	0.6804 (3)	1.0640 (4)	0.0494 (4)	0.263 (3)
H53'	0.6925	0.6531	1.0782	0.059*	0.263 (3)

### Atomic displacement parameters (Å^2^)

**Table:**

	*U*^11^	*U*^22^	*U*^33^	*U*^12^	*U*^13^	*U*^23^
Ni	0.02219 (14)	0.01680 (13)	0.01589 (12)	−0.00195 (10)	0.00117 (9)	−0.00103 (9)
N1	0.0220 (9)	0.0225 (8)	0.0201 (8)	0.0003 (7)	0.0004 (6)	−0.0026 (7)
N2	0.0236 (10)	0.0395 (11)	0.0256 (9)	0.0061 (8)	−0.0004 (7)	−0.0083 (8)
N3	0.0248 (9)	0.0162 (8)	0.0183 (8)	−0.0010 (7)	0.0005 (6)	−0.0004 (6)
N4	0.0239 (9)	0.0162 (8)	0.0184 (8)	−0.0018 (7)	−0.0016 (6)	−0.0010 (6)
N5	0.0253 (10)	0.0207 (9)	0.0246 (9)	0.0006 (7)	−0.0030 (7)	−0.0077 (7)
N6	0.0230 (9)	0.0182 (8)	0.0165 (8)	−0.0023 (7)	0.0027 (6)	−0.0008 (6)
N7	0.0248 (9)	0.0163 (8)	0.0180 (8)	−0.0013 (7)	0.0035 (6)	−0.0023 (6)
N8	0.0220 (9)	0.0202 (8)	0.0156 (7)	−0.0029 (7)	0.0014 (6)	−0.0013 (6)
N9	0.0251 (9)	0.0206 (8)	0.0206 (8)	−0.0026 (7)	0.0000 (7)	0.0022 (7)
N10	0.0286 (10)	0.0312 (10)	0.0205 (8)	−0.0002 (8)	0.0047 (7)	0.0069 (7)
C1	0.0228 (11)	0.0266 (11)	0.0210 (9)	−0.0021 (8)	−0.0006 (8)	−0.0032 (8)
C2	0.0249 (11)	0.0306 (11)	0.0243 (10)	0.0008 (9)	0.0007 (8)	−0.0033 (9)
C3	0.0325 (13)	0.0413 (13)	0.0227 (10)	−0.0031 (10)	−0.0012 (9)	−0.0092 (9)
C4	0.0284 (13)	0.0565 (16)	0.0270 (11)	0.0029 (11)	−0.0071 (9)	−0.0090 (11)
C5	0.0255 (12)	0.0571 (16)	0.0346 (12)	0.0092 (11)	−0.0039 (10)	−0.0099 (11)
C6	0.0249 (11)	0.0366 (12)	0.0239 (10)	0.0005 (9)	−0.0012 (8)	−0.0076 (9)
C7	0.0216 (11)	0.0240 (10)	0.0219 (10)	−0.0005 (8)	0.0018 (8)	−0.0005 (8)
C8	0.0251 (11)	0.0189 (10)	0.0211 (9)	−0.0008 (8)	0.0021 (8)	0.0011 (8)
C9	0.0240 (11)	0.0235 (10)	0.0252 (10)	−0.0009 (8)	0.0033 (8)	−0.0010 (8)
C10	0.0311 (12)	0.0217 (10)	0.0201 (9)	−0.0003 (9)	0.0046 (8)	−0.0029 (8)
C11	0.0290 (12)	0.0208 (10)	0.0200 (9)	−0.0010 (8)	−0.0005 (8)	−0.0021 (8)
C12	0.0247 (11)	0.0164 (9)	0.0188 (9)	−0.0005 (8)	−0.0008 (7)	0.0004 (7)
C13	0.0258 (11)	0.0169 (9)	0.0169 (9)	0.0003 (8)	−0.0015 (7)	−0.0007 (7)
C14	0.0261 (11)	0.0191 (10)	0.0226 (10)	−0.0002 (8)	−0.0025 (8)	−0.0012 (8)
C15	0.0278 (12)	0.0302 (11)	0.0294 (11)	−0.0014 (9)	−0.0051 (9)	−0.0067 (9)
C16	0.0226 (11)	0.0379 (12)	0.0268 (11)	0.0017 (9)	−0.0026 (8)	0.0019 (9)
C17	0.0301 (12)	0.0280 (11)	0.0202 (10)	0.0045 (9)	0.0034 (8)	0.0021 (8)
C18	0.0308 (12)	0.0210 (10)	0.0169 (9)	−0.0007 (8)	0.0015 (8)	0.0005 (8)
C19	0.0250 (11)	0.0170 (9)	0.0184 (9)	−0.0002 (8)	−0.0012 (7)	0.0018 (7)
C20	0.0209 (10)	0.0205 (10)	0.0192 (9)	−0.0020 (8)	0.0014 (7)	0.0006 (8)
C21	0.0330 (12)	0.0205 (10)	0.0191 (9)	−0.0010 (9)	0.0021 (8)	−0.0028 (8)
C22	0.0325 (12)	0.0262 (11)	0.0192 (10)	0.0029 (9)	0.0053 (8)	−0.0012 (8)
C23	0.0306 (12)	0.0270 (11)	0.0204 (10)	−0.0005 (9)	0.0055 (8)	0.0029 (8)
C24	0.0281 (11)	0.0207 (10)	0.0229 (10)	−0.0018 (8)	0.0043 (8)	0.0023 (8)
C25	0.0208 (10)	0.0204 (9)	0.0170 (9)	−0.0005 (8)	−0.0003 (7)	−0.0018 (7)
C26	0.0204 (10)	0.0187 (9)	0.0177 (9)	−0.0017 (7)	0.0002 (7)	−0.0011 (7)
C27	0.0195 (10)	0.0219 (10)	0.0180 (9)	−0.0034 (8)	0.0012 (7)	−0.0013 (7)
C28	0.0257 (11)	0.0195 (10)	0.0216 (9)	−0.0027 (8)	0.0011 (8)	−0.0021 (8)
C29	0.0308 (12)	0.0267 (11)	0.0190 (9)	0.0003 (9)	0.0005 (8)	−0.0061 (8)
C30	0.0283 (12)	0.0313 (11)	0.0178 (9)	−0.0015 (9)	0.0024 (8)	−0.0016 (8)
C31	0.0225 (11)	0.0243 (10)	0.0167 (9)	−0.0016 (8)	0.0008 (7)	0.0010 (8)
C32	0.0220 (10)	0.0294 (11)	0.0150 (9)	−0.0035 (8)	−0.0005 (7)	0.0034 (8)
C33	0.0245 (12)	0.0260 (11)	0.0329 (11)	0.0019 (9)	0.0027 (9)	0.0109 (9)
C34	0.0414 (15)	0.0315 (13)	0.0588 (16)	0.0066 (11)	0.0223 (12)	0.0217 (12)
C35	0.0474 (17)	0.0239 (13)	0.094 (2)	0.0018 (11)	0.0305 (16)	0.0186 (14)
C36	0.0399 (15)	0.0204 (12)	0.0781 (19)	0.0024 (10)	0.0140 (13)	0.0025 (12)
C37	0.0293 (13)	0.0219 (11)	0.0451 (13)	0.0000 (9)	0.0026 (10)	0.0033 (9)
C38	0.0218 (11)	0.0227 (10)	0.0308 (11)	0.0013 (8)	−0.0016 (8)	0.0092 (8)
O1	0.0415 (10)	0.0212 (8)	0.0284 (8)	−0.0015 (7)	−0.0020 (7)	0.0066 (6)
O2	0.0639 (13)	0.0310 (9)	0.0270 (8)	−0.0065 (8)	−0.0049 (8)	0.0090 (7)
O3	0.0555 (12)	0.0365 (9)	0.0293 (8)	−0.0049 (8)	0.0113 (8)	−0.0024 (7)
O4	0.0396 (10)	0.0256 (8)	0.0398 (9)	−0.0052 (7)	0.0011 (7)	0.0010 (7)
O5	0.0525 (12)	0.0335 (9)	0.0349 (9)	−0.0056 (8)	0.0030 (8)	0.0141 (7)
O6	0.0740 (15)	0.0495 (11)	0.0258 (9)	0.0124 (10)	−0.0063 (8)	−0.0024 (8)
O7	0.0587 (12)	0.0321 (9)	0.0459 (10)	0.0147 (8)	0.0166 (9)	0.0028 (8)
N11	0.0336 (11)	0.0282 (10)	0.0261 (9)	−0.0085 (8)	−0.0004 (8)	0.0037 (8)
N12	0.0258 (10)	0.0268 (10)	0.0350 (10)	−0.0025 (8)	0.0017 (8)	0.0096 (8)
N13	0.0301 (11)	0.0321 (10)	0.0304 (10)	0.0032 (8)	0.0066 (8)	0.0009 (8)
C39	0.0198 (11)	0.0252 (11)	0.0269 (10)	0.0019 (8)	−0.0003 (8)	0.0061 (8)
C40	0.0240 (11)	0.0263 (11)	0.0218 (10)	−0.0032 (8)	0.0001 (8)	0.0055 (8)
C41	0.0219 (11)	0.0269 (11)	0.0264 (10)	−0.0028 (8)	−0.0003 (8)	0.0013 (9)
C42	0.0225 (11)	0.0230 (10)	0.0297 (11)	−0.0027 (8)	0.0007 (8)	0.0071 (8)
C43	0.0218 (11)	0.0300 (11)	0.0260 (10)	0.0001 (9)	0.0025 (8)	0.0088 (9)
C44	0.0214 (11)	0.0260 (11)	0.0246 (10)	0.0020 (8)	0.0026 (8)	0.0021 (8)
O8	0.0454 (10)	0.0187 (7)	0.0262 (8)	−0.0029 (7)	0.0034 (7)	−0.0021 (6)
O9	0.0354 (9)	0.0271 (8)	0.0260 (7)	−0.0073 (7)	0.0045 (6)	0.0026 (6)
O10	0.0389 (10)	0.0425 (9)	0.0220 (7)	−0.0096 (7)	−0.0042 (6)	−0.0048 (7)
O11	0.0667 (13)	0.0230 (8)	0.0463 (10)	−0.0078 (8)	−0.0019 (9)	−0.0069 (8)
O12	0.0429 (11)	0.0315 (9)	0.0438 (10)	−0.0112 (8)	0.0002 (8)	0.0125 (7)
O13	0.0507 (11)	0.0512 (11)	0.0217 (8)	−0.0048 (8)	0.0104 (7)	−0.0016 (7)
O14	0.0414 (10)	0.0280 (8)	0.0311 (8)	−0.0033 (7)	−0.0014 (7)	−0.0070 (6)
N14	0.0222 (9)	0.0250 (9)	0.0228 (8)	−0.0004 (7)	0.0006 (7)	−0.0006 (7)
N15	0.0299 (11)	0.0218 (9)	0.0416 (11)	−0.0046 (8)	−0.0046 (8)	0.0022 (8)
N16	0.0279 (10)	0.0278 (9)	0.0219 (8)	0.0071 (8)	0.0020 (7)	−0.0008 (7)
C45	0.0194 (10)	0.0198 (10)	0.0239 (10)	−0.0011 (8)	0.0029 (8)	−0.0015 (8)
C46	0.0210 (10)	0.0210 (10)	0.0203 (9)	−0.0003 (8)	0.0019 (7)	0.0010 (8)
C47	0.0197 (10)	0.0239 (10)	0.0253 (10)	−0.0004 (8)	−0.0005 (8)	−0.0046 (8)
C48	0.0222 (11)	0.0182 (10)	0.0327 (11)	−0.0013 (8)	0.0004 (8)	0.0021 (8)
C49	0.0210 (11)	0.0255 (10)	0.0239 (10)	0.0019 (8)	0.0033 (8)	0.0036 (8)
C50	0.0210 (11)	0.0229 (10)	0.0194 (9)	−0.0002 (8)	0.0010 (7)	−0.0029 (8)
O16	0.0338 (10)	0.0559 (11)	0.0443 (10)	−0.0117 (8)	0.0048 (8)	0.0139 (8)
N18	0.0356 (11)	0.0314 (10)	0.0258 (9)	−0.0022 (8)	0.0030 (8)	0.0036 (8)
C54	0.088 (2)	0.0507 (17)	0.0353 (14)	−0.0159 (16)	−0.0174 (14)	0.0164 (13)
C55	0.0409 (16)	0.0618 (18)	0.0374 (13)	−0.0176 (13)	0.0032 (11)	0.0024 (12)
C56	0.0298 (13)	0.0406 (13)	0.0325 (12)	0.0006 (10)	0.0074 (10)	0.0092 (10)
O15	0.0400 (10)	0.0783 (11)	0.0301 (6)	−0.0152 (7)	0.0030 (6)	0.0053 (6)
N17	0.0400 (10)	0.0783 (11)	0.0301 (6)	−0.0152 (7)	0.0030 (6)	0.0053 (6)
C51	0.0400 (10)	0.0783 (11)	0.0301 (6)	−0.0152 (7)	0.0030 (6)	0.0053 (6)
C52	0.0400 (10)	0.0783 (11)	0.0301 (6)	−0.0152 (7)	0.0030 (6)	0.0053 (6)
C53	0.0400 (10)	0.0783 (11)	0.0301 (6)	−0.0152 (7)	0.0030 (6)	0.0053 (6)
O15'	0.0400 (10)	0.0783 (11)	0.0301 (6)	−0.0152 (7)	0.0030 (6)	0.0053 (6)
N17'	0.0400 (10)	0.0783 (11)	0.0301 (6)	−0.0152 (7)	0.0030 (6)	0.0053 (6)
C51'	0.0400 (10)	0.0783 (11)	0.0301 (6)	−0.0152 (7)	0.0030 (6)	0.0053 (6)
C52'	0.0400 (10)	0.0783 (11)	0.0301 (6)	−0.0152 (7)	0.0030 (6)	0.0053 (6)
C53'	0.0400 (10)	0.0783 (11)	0.0301 (6)	−0.0152 (7)	0.0030 (6)	0.0053 (6)

### Geometric parameters (Å, °)

**Table:**

Ni—N8	2.0393 (16)	C33—C38	1.405 (3)
Ni—N3	2.0469 (16)	C34—C35	1.373 (4)
Ni—N9	2.1059 (16)	C34—H34A	0.9500
Ni—N6	2.1128 (16)	C35—C36	1.398 (4)
Ni—N1	2.1134 (17)	C35—H35A	0.9500
Ni—N4	2.1182 (16)	C36—C37	1.377 (3)
N1—C7	1.328 (3)	C36—H36A	0.9500
N1—C1	1.387 (2)	C37—C38	1.397 (3)
N2—C7	1.351 (3)	C37—H37A	0.9500
N2—C6	1.390 (3)	O1—C39	1.249 (2)
N2—H2B	0.8800	O2—N11	1.230 (2)
N3—C8	1.337 (3)	O3—N11	1.228 (2)
N3—C12	1.339 (2)	O4—N12	1.227 (2)
N4—C13	1.330 (2)	O5—N12	1.238 (2)
N4—C19	1.380 (3)	O6—N13	1.230 (2)
N5—C13	1.345 (3)	O7—N13	1.227 (2)
N5—C14	1.380 (3)	N11—C40	1.454 (3)
N5—H5B	0.8800	N12—C42	1.449 (3)
N6—C26	1.327 (2)	N13—C44	1.453 (3)
N6—C20	1.385 (2)	C39—C44	1.449 (3)
N7—C26	1.351 (2)	C39—C40	1.451 (3)
N7—C25	1.385 (2)	C40—C41	1.370 (3)
N7—H7A	0.8800	C41—C42	1.390 (3)
N8—C27	1.334 (2)	C41—H41A	0.9500
N8—C31	1.343 (2)	C42—C43	1.378 (3)
N9—C32	1.327 (3)	C43—C44	1.369 (3)
N9—C38	1.378 (3)	C43—H43A	0.9500
N10—C32	1.348 (2)	O8—C45	1.243 (2)
N10—C33	1.375 (3)	O9—N14	1.229 (2)
N10—H10B	0.8800	O10—N14	1.233 (2)
C1—C2	1.392 (3)	O11—N15	1.226 (2)
C1—C6	1.401 (3)	O12—N15	1.235 (2)
C2—C3	1.378 (3)	O13—N16	1.235 (2)
C2—H2A	0.9500	O14—N16	1.224 (2)
C3—C4	1.402 (3)	N14—C46	1.459 (2)
C3—H3A	0.9500	N15—C48	1.446 (3)
C4—C5	1.377 (3)	N16—C50	1.453 (2)
C4—H4A	0.9500	C45—C50	1.455 (3)
C5—C6	1.387 (3)	C45—C46	1.455 (3)
C5—H5A	0.9500	C46—C47	1.368 (3)
C7—C8	1.466 (3)	C47—C48	1.388 (3)
C8—C9	1.387 (3)	C47—H47A	0.9500
C9—C10	1.396 (3)	C48—C49	1.382 (3)
C9—H9A	0.9500	C49—C50	1.362 (3)
C10—C11	1.380 (3)	C49—H49A	0.9500
C10—H10A	0.9500	O16—C56	1.241 (3)
C11—C12	1.400 (3)	N18—C56	1.314 (3)
C11—H11A	0.9500	N18—C54	1.445 (3)
C12—C13	1.457 (3)	N18—C55	1.454 (3)
C14—C15	1.387 (3)	C54—H54A	0.9800
C14—C19	1.408 (3)	C54—H54B	0.9800
C15—C16	1.375 (3)	C54—H54C	0.9800
C15—H15A	0.9500	C55—H55A	0.9800
C16—C17	1.406 (3)	C55—H55B	0.9800
C16—H16A	0.9500	C55—H55C	0.9800
C17—C18	1.378 (3)	C56—H56	0.9500
C17—H17A	0.9500	O15—C53	1.230 (4)
C18—C19	1.399 (3)	N17—C53	1.317 (4)
C18—H18A	0.9500	N17—C51	1.440 (4)
C20—C21	1.399 (3)	N17—C52	1.445 (3)
C20—C25	1.400 (3)	C51—H51A	0.9800
C21—C22	1.374 (3)	C51—H51B	0.9800
C21—H21A	0.9500	C51—H51C	0.9800
C22—C23	1.398 (3)	C52—H52A	0.9800
C22—H22A	0.9500	C52—H52B	0.9800
C23—C24	1.380 (3)	C52—H52C	0.9800
C23—H23A	0.9500	C53—H53	0.9500
C24—C25	1.388 (3)	O15'—C53'	1.223 (6)
C24—H24A	0.9500	N17'—C53'	1.327 (5)
C26—C27	1.466 (3)	N17'—C52'	1.431 (4)
C27—C28	1.394 (3)	N17'—C51'	1.444 (4)
C28—C29	1.385 (3)	C51'—H51D	0.9800
C28—H28A	0.9500	C51'—H51E	0.9800
C29—C30	1.386 (3)	C51'—H51F	0.9800
C29—H29A	0.9500	C52'—H52D	0.9800
C30—C31	1.384 (3)	C52'—H52E	0.9800
C30—H30A	0.9500	C52'—H52F	0.9800
C31—C32	1.461 (3)	C53'—H53'	0.9500
C33—C34	1.390 (3)		
			
N8—Ni—N3	171.79 (7)	C29—C30—H30A	121.2
N8—Ni—N9	77.21 (6)	N8—C31—C30	121.77 (18)
N3—Ni—N9	110.07 (6)	N8—C31—C32	110.29 (17)
N8—Ni—N6	77.17 (6)	C30—C31—C32	127.93 (18)
N3—Ni—N6	95.78 (6)	N9—C32—N10	113.13 (18)
N9—Ni—N6	154.04 (6)	N9—C32—C31	119.69 (17)
N8—Ni—N1	99.23 (6)	N10—C32—C31	126.95 (18)
N3—Ni—N1	76.69 (6)	N10—C33—C34	131.7 (2)
N9—Ni—N1	95.38 (6)	N10—C33—C38	106.15 (18)
N6—Ni—N1	92.78 (6)	C34—C33—C38	122.1 (2)
N8—Ni—N4	107.44 (6)	C35—C34—C33	116.0 (2)
N3—Ni—N4	77.22 (6)	C35—C34—H34A	122.0
N9—Ni—N4	87.60 (6)	C33—C34—H34A	122.0
N6—Ni—N4	96.09 (6)	C34—C35—C36	122.8 (2)
N1—Ni—N4	153.14 (6)	C34—C35—H35A	118.6
C7—N1—C1	105.07 (17)	C36—C35—H35A	118.6
C7—N1—Ni	112.91 (13)	C37—C36—C35	121.3 (2)
C1—N1—Ni	141.64 (14)	C37—C36—H36A	119.4
C7—N2—C6	106.87 (17)	C35—C36—H36A	119.4
C7—N2—H2B	126.6	C36—C37—C38	117.1 (2)
C6—N2—H2B	126.6	C36—C37—H37A	121.5
C8—N3—C12	120.94 (17)	C38—C37—H37A	121.5
C8—N3—Ni	119.13 (13)	N9—C38—C37	130.4 (2)
C12—N3—Ni	118.83 (13)	N9—C38—C33	108.83 (18)
C13—N4—C19	104.88 (16)	C37—C38—C33	120.7 (2)
C13—N4—Ni	112.17 (13)	O3—N11—O2	123.00 (18)
C19—N4—Ni	139.12 (13)	O3—N11—C40	118.66 (18)
C13—N5—C14	106.56 (16)	O2—N11—C40	118.32 (18)
C13—N5—H5B	126.7	O4—N12—O5	123.48 (18)
C14—N5—H5B	126.7	O4—N12—C42	118.91 (17)
C26—N6—C20	105.18 (15)	O5—N12—C42	117.61 (18)
C26—N6—Ni	112.83 (12)	O7—N13—O6	122.71 (19)
C20—N6—Ni	141.95 (13)	O7—N13—C44	118.90 (18)
C26—N7—C25	106.70 (15)	O6—N13—C44	118.38 (18)
C26—N7—H7A	126.6	O1—C39—C44	122.38 (19)
C25—N7—H7A	126.6	O1—C39—C40	125.86 (19)
C27—N8—C31	120.38 (16)	C44—C39—C40	111.62 (18)
C27—N8—Ni	119.77 (13)	C41—C40—C39	124.21 (18)
C31—N8—Ni	119.65 (13)	C41—C40—N11	116.26 (18)
C32—N9—C38	105.31 (17)	C39—C40—N11	119.48 (17)
C32—N9—Ni	112.81 (13)	C40—C41—C42	119.15 (19)
C38—N9—Ni	141.58 (14)	C40—C41—H41A	120.4
C32—N10—C33	106.56 (17)	C42—C41—H41A	120.4
C32—N10—H10B	126.7	C43—C42—C41	121.16 (19)
C33—N10—H10B	126.7	C43—C42—N12	119.05 (18)
N1—C1—C2	130.0 (2)	C41—C42—N12	119.79 (19)
N1—C1—C6	109.50 (17)	C44—C43—C42	119.17 (19)
C2—C1—C6	120.40 (19)	C44—C43—H43A	120.4
C3—C2—C1	117.6 (2)	C42—C43—H43A	120.4
C3—C2—H2A	121.2	C43—C44—C39	124.58 (19)
C1—C2—H2A	121.2	C43—C44—N13	116.60 (18)
C2—C3—C4	121.2 (2)	C39—C44—N13	118.79 (18)
C2—C3—H3A	119.4	O9—N14—O10	122.78 (17)
C4—C3—H3A	119.4	O9—N14—C46	119.29 (16)
C5—C4—C3	122.1 (2)	O10—N14—C46	117.90 (17)
C5—C4—H4A	119.0	O11—N15—O12	123.62 (18)
C3—C4—H4A	119.0	O11—N15—C48	118.32 (19)
C4—C5—C6	116.4 (2)	O12—N15—C48	118.06 (18)
C4—C5—H5A	121.8	O14—N16—O13	122.81 (18)
C6—C5—H5A	121.8	O14—N16—C50	119.39 (17)
C5—C6—N2	132.2 (2)	O13—N16—C50	117.77 (18)
C5—C6—C1	122.3 (2)	O8—C45—C50	124.05 (18)
N2—C6—C1	105.43 (18)	O8—C45—C46	124.57 (18)
N1—C7—N2	113.08 (17)	C50—C45—C46	111.30 (17)
N1—C7—C8	119.06 (18)	C47—C46—C45	124.36 (18)
N2—C7—C8	127.50 (18)	C47—C46—N14	116.13 (17)
N3—C8—C9	121.59 (18)	C45—C46—N14	119.50 (17)
N3—C8—C7	110.23 (17)	C46—C47—C48	119.14 (18)
C9—C8—C7	128.18 (19)	C46—C47—H47A	120.4
C8—C9—C10	117.5 (2)	C48—C47—H47A	120.4
C8—C9—H9A	121.2	C49—C48—C47	120.98 (18)
C10—C9—H9A	121.2	C49—C48—N15	119.00 (18)
C11—C10—C9	120.99 (19)	C47—C48—N15	120.02 (18)
C11—C10—H10A	119.5	C50—C49—C48	119.48 (18)
C9—C10—H10A	119.5	C50—C49—H49A	120.3
C10—C11—C12	117.86 (18)	C48—C49—H49A	120.3
C10—C11—H11A	121.1	C49—C50—N16	116.37 (17)
C12—C11—H11A	121.1	C49—C50—C45	124.41 (18)
N3—C12—C11	120.99 (19)	N16—C50—C45	119.22 (17)
N3—C12—C13	110.74 (16)	C56—N18—C54	121.2 (2)
C11—C12—C13	128.26 (18)	C56—N18—C55	122.2 (2)
N4—C13—N5	113.55 (18)	C54—N18—C55	116.5 (2)
N4—C13—C12	118.88 (17)	N18—C54—H54A	109.5
N5—C13—C12	127.42 (17)	N18—C54—H54B	109.5
N5—C14—C15	131.81 (18)	H54A—C54—H54B	109.5
N5—C14—C19	105.83 (18)	N18—C54—H54C	109.5
C15—C14—C19	122.36 (19)	H54A—C54—H54C	109.5
C16—C15—C14	116.38 (19)	H54B—C54—H54C	109.5
C16—C15—H15A	121.8	N18—C55—H55A	109.5
C14—C15—H15A	121.8	N18—C55—H55B	109.5
C15—C16—C17	122.1 (2)	H55A—C55—H55B	109.5
C15—C16—H16A	119.0	N18—C55—H55C	109.5
C17—C16—H16A	119.0	H55A—C55—H55C	109.5
C18—C17—C16	121.7 (2)	H55B—C55—H55C	109.5
C18—C17—H17A	119.1	O16—C56—N18	125.9 (2)
C16—C17—H17A	119.1	O16—C56—H56	117.1
C17—C18—C19	116.92 (18)	N18—C56—H56	117.1
C17—C18—H18A	121.5	C53—N17—C51	122.2 (2)
C19—C18—H18A	121.5	C53—N17—C52	120.5 (3)
N4—C19—C18	130.30 (18)	C51—N17—C52	117.1 (3)
N4—C19—C14	109.18 (17)	N17—C51—H51A	109.5
C18—C19—C14	120.52 (19)	N17—C51—H51B	109.5
N6—C20—C21	130.26 (18)	H51A—C51—H51B	109.5
N6—C20—C25	109.25 (16)	N17—C51—H51C	109.5
C21—C20—C25	120.49 (17)	H51A—C51—H51C	109.5
C22—C21—C20	117.37 (19)	H51B—C51—H51C	109.5
C22—C21—H21A	121.3	N17—C52—H52A	109.5
C20—C21—H21A	121.3	N17—C52—H52B	109.5
C21—C22—C23	121.50 (19)	H52A—C52—H52B	109.5
C21—C22—H22A	119.2	N17—C52—H52C	109.5
C23—C22—H22A	119.2	H52A—C52—H52C	109.5
C24—C23—C22	122.05 (19)	H52B—C52—H52C	109.5
C24—C23—H23A	119.0	O15—C53—N17	125.7 (3)
C22—C23—H23A	119.0	O15—C53—H53	117.1
C23—C24—C25	116.48 (19)	N17—C53—H53	117.1
C23—C24—H24A	121.8	C53'—N17'—C52'	121.7 (3)
C25—C24—H24A	121.8	C53'—N17'—C51'	119.6 (4)
N7—C25—C24	132.11 (18)	C52'—N17'—C51'	118.7 (4)
N7—C25—C20	105.80 (16)	N17'—C51'—H51D	109.5
C24—C25—C20	122.09 (18)	N17'—C51'—H51E	109.5
N6—C26—N7	113.06 (16)	H51D—C51'—H51E	109.5
N6—C26—C27	119.17 (17)	N17'—C51'—H51F	109.5
N7—C26—C27	127.76 (17)	H51D—C51'—H51F	109.5
N8—C27—C28	121.57 (17)	H51E—C51'—H51F	109.5
N8—C27—C26	110.80 (16)	N17'—C52'—H52D	109.5
C28—C27—C26	127.63 (18)	N17'—C52'—H52E	109.5
C29—C28—C27	117.55 (18)	H52D—C52'—H52E	109.5
C29—C28—H28A	121.2	N17'—C52'—H52F	109.5
C27—C28—H28A	121.2	H52D—C52'—H52F	109.5
C28—C29—C30	121.10 (19)	H52E—C52'—H52F	109.5
C28—C29—H29A	119.4	O15'—C53'—N17'	124.8 (6)
C30—C29—H29A	119.4	O15'—C53'—H53'	117.6
C31—C30—C29	117.62 (18)	N17'—C53'—H53'	117.6
C31—C30—H30A	121.2		
			
N8—Ni—N1—C7	165.53 (14)	Ni—N6—C20—C21	4.8 (4)
N3—Ni—N1—C7	−7.21 (13)	C26—N6—C20—C25	0.9 (2)
N9—Ni—N1—C7	−116.59 (14)	Ni—N6—C20—C25	−176.29 (16)
N6—Ni—N1—C7	88.08 (14)	N6—C20—C21—C22	179.0 (2)
N4—Ni—N1—C7	−21.3 (2)	C25—C20—C21—C22	0.2 (3)
N8—Ni—N1—C1	−22.9 (2)	C20—C21—C22—C23	0.5 (3)
N3—Ni—N1—C1	164.4 (2)	C21—C22—C23—C24	−0.4 (3)
N9—Ni—N1—C1	55.0 (2)	C22—C23—C24—C25	−0.4 (3)
N6—Ni—N1—C1	−100.3 (2)	C26—N7—C25—C24	179.4 (2)
N4—Ni—N1—C1	150.32 (19)	C26—N7—C25—C20	−0.2 (2)
N8—Ni—N3—C8	−47.8 (5)	C23—C24—C25—N7	−178.4 (2)
N9—Ni—N3—C8	104.03 (14)	C23—C24—C25—C20	1.1 (3)
N6—Ni—N3—C8	−78.39 (14)	N6—C20—C25—N7	−0.4 (2)
N1—Ni—N3—C8	13.11 (14)	C21—C20—C25—N7	178.61 (18)
N4—Ni—N3—C8	−173.35 (15)	N6—C20—C25—C24	179.88 (18)
N8—Ni—N3—C12	120.3 (4)	C21—C20—C25—C24	−1.1 (3)
N9—Ni—N3—C12	−87.88 (15)	C20—N6—C26—N7	−1.1 (2)
N6—Ni—N3—C12	89.70 (14)	Ni—N6—C26—N7	177.05 (13)
N1—Ni—N3—C12	−178.81 (15)	C20—N6—C26—C27	179.55 (17)
N4—Ni—N3—C12	−5.26 (14)	Ni—N6—C26—C27	−2.3 (2)
N8—Ni—N4—C13	−177.33 (13)	C25—N7—C26—N6	0.8 (2)
N3—Ni—N4—C13	−4.33 (13)	C25—N7—C26—C27	−179.87 (19)
N9—Ni—N4—C13	106.88 (14)	C31—N8—C27—C28	−0.8 (3)
N6—Ni—N4—C13	−98.90 (13)	Ni—N8—C27—C28	174.11 (15)
N1—Ni—N4—C13	9.7 (2)	C31—N8—C27—C26	179.45 (17)
N8—Ni—N4—C19	29.2 (2)	Ni—N8—C27—C26	−5.6 (2)
N3—Ni—N4—C19	−157.8 (2)	N6—C26—C27—N8	5.1 (3)
N9—Ni—N4—C19	−46.57 (19)	N7—C26—C27—N8	−174.14 (19)
N6—Ni—N4—C19	107.65 (19)	N6—C26—C27—C28	−174.60 (19)
N1—Ni—N4—C19	−143.76 (18)	N7—C26—C27—C28	6.1 (3)
N8—Ni—N6—C26	−0.53 (13)	N8—C27—C28—C29	1.4 (3)
N3—Ni—N6—C26	175.20 (14)	C26—C27—C28—C29	−178.93 (19)
N9—Ni—N6—C26	−10.0 (2)	C27—C28—C29—C30	−0.8 (3)
N1—Ni—N6—C26	98.31 (14)	C28—C29—C30—C31	−0.2 (3)
N4—Ni—N6—C26	−107.08 (14)	C27—N8—C31—C30	−0.3 (3)
N8—Ni—N6—C20	176.5 (2)	Ni—N8—C31—C30	−175.26 (15)
N3—Ni—N6—C20	−7.7 (2)	C27—N8—C31—C32	−179.12 (17)
N9—Ni—N6—C20	167.07 (19)	Ni—N8—C31—C32	6.0 (2)
N1—Ni—N6—C20	−84.6 (2)	C29—C30—C31—N8	0.8 (3)
N4—Ni—N6—C20	70.0 (2)	C29—C30—C31—C32	179.4 (2)
N3—Ni—N8—C27	−27.6 (5)	C38—N9—C32—N10	−1.8 (2)
N9—Ni—N8—C27	179.42 (16)	Ni—N9—C32—N10	−176.88 (13)
N6—Ni—N8—C27	3.66 (14)	C38—N9—C32—C31	173.06 (18)
N1—Ni—N8—C27	−87.11 (15)	Ni—N9—C32—C31	−2.1 (2)
N4—Ni—N8—C27	96.10 (15)	C33—N10—C32—N9	1.3 (2)
N3—Ni—N8—C31	147.3 (4)	C33—N10—C32—C31	−173.1 (2)
N9—Ni—N8—C31	−5.64 (14)	N8—C31—C32—N9	−2.3 (3)
N6—Ni—N8—C31	178.60 (16)	C30—C31—C32—N9	179.0 (2)
N1—Ni—N8—C31	87.83 (15)	N8—C31—C32—N10	171.71 (19)
N4—Ni—N8—C31	−88.96 (15)	C30—C31—C32—N10	−7.0 (3)
N8—Ni—N9—C32	3.86 (14)	C32—N10—C33—C34	177.4 (2)
N3—Ni—N9—C32	−172.18 (13)	C32—N10—C33—C38	−0.2 (2)
N6—Ni—N9—C32	13.3 (2)	N10—C33—C34—C35	−174.7 (2)
N1—Ni—N9—C32	−94.40 (14)	C38—C33—C34—C35	2.6 (4)
N4—Ni—N9—C32	112.35 (14)	C33—C34—C35—C36	−1.7 (4)
N8—Ni—N9—C38	−168.6 (2)	C34—C35—C36—C37	−0.3 (5)
N3—Ni—N9—C38	15.4 (2)	C35—C36—C37—C38	1.4 (4)
N6—Ni—N9—C38	−159.09 (19)	C32—N9—C38—C37	−175.3 (2)
N1—Ni—N9—C38	93.2 (2)	Ni—N9—C38—C37	−2.5 (4)
N4—Ni—N9—C38	−60.1 (2)	C32—N9—C38—C33	1.5 (2)
C7—N1—C1—C2	177.6 (2)	Ni—N9—C38—C33	174.30 (17)
Ni—N1—C1—C2	5.7 (4)	C36—C37—C38—N9	176.0 (2)
C7—N1—C1—C6	0.7 (2)	C36—C37—C38—C33	−0.5 (3)
Ni—N1—C1—C6	−171.22 (17)	N10—C33—C38—N9	−0.8 (2)
N1—C1—C2—C3	−174.9 (2)	C34—C33—C38—N9	−178.8 (2)
C6—C1—C2—C3	1.7 (3)	N10—C33—C38—C37	176.37 (19)
C1—C2—C3—C4	−0.4 (3)	C34—C33—C38—C37	−1.6 (3)
C2—C3—C4—C5	−1.0 (4)	O1—C39—C40—C41	172.0 (2)
C3—C4—C5—C6	1.0 (4)	C44—C39—C40—C41	−3.7 (3)
C4—C5—C6—N2	176.8 (2)	O1—C39—C40—N11	−5.5 (3)
C4—C5—C6—C1	0.4 (4)	C44—C39—C40—N11	178.80 (18)
C7—N2—C6—C5	−174.8 (3)	O3—N11—C40—C41	−23.0 (3)
C7—N2—C6—C1	2.0 (2)	O2—N11—C40—C41	155.6 (2)
N1—C1—C6—C5	175.5 (2)	O3—N11—C40—C39	154.8 (2)
C2—C1—C6—C5	−1.7 (3)	O2—N11—C40—C39	−26.7 (3)
N1—C1—C6—N2	−1.7 (2)	C39—C40—C41—C42	2.2 (3)
C2—C1—C6—N2	−178.97 (19)	N11—C40—C41—C42	179.79 (19)
C1—N1—C7—N2	0.6 (2)	C40—C41—C42—C43	−0.5 (3)
Ni—N1—C7—N2	175.19 (14)	C40—C41—C42—N12	178.68 (19)
C1—N1—C7—C8	−173.09 (17)	O4—N12—C42—C43	173.3 (2)
Ni—N1—C7—C8	1.5 (2)	O5—N12—C42—C43	−6.1 (3)
C6—N2—C7—N1	−1.7 (2)	O4—N12—C42—C41	−6.0 (3)
C6—N2—C7—C8	171.3 (2)	O5—N12—C42—C41	174.7 (2)
C12—N3—C8—C9	−3.1 (3)	C41—C42—C43—C44	0.8 (3)
Ni—N3—C8—C9	164.76 (15)	N12—C42—C43—C44	−178.42 (19)
C12—N3—C8—C7	176.67 (17)	C42—C43—C44—C39	−2.8 (3)
Ni—N3—C8—C7	−15.5 (2)	C42—C43—C44—N13	179.34 (19)
N1—C7—C8—N3	8.7 (3)	O1—C39—C44—C43	−171.9 (2)
N2—C7—C8—N3	−164.0 (2)	C40—C39—C44—C43	4.0 (3)
N1—C7—C8—C9	−171.6 (2)	O1—C39—C44—N13	6.0 (3)
N2—C7—C8—C9	15.8 (3)	C40—C39—C44—N13	−178.16 (18)
N3—C8—C9—C10	1.2 (3)	O7—N13—C44—C43	−147.2 (2)
C7—C8—C9—C10	−178.52 (19)	O6—N13—C44—C43	31.6 (3)
C8—C9—C10—C11	1.7 (3)	O7—N13—C44—C39	34.8 (3)
C9—C10—C11—C12	−2.7 (3)	O6—N13—C44—C39	−146.4 (2)
C8—N3—C12—C11	2.0 (3)	O8—C45—C46—C47	−171.9 (2)
Ni—N3—C12—C11	−165.83 (14)	C50—C45—C46—C47	5.0 (3)
C8—N3—C12—C13	−179.33 (16)	O8—C45—C46—N14	6.9 (3)
Ni—N3—C12—C13	12.8 (2)	C50—C45—C46—N14	−176.16 (17)
C10—C11—C12—N3	0.8 (3)	O9—N14—C46—C47	−156.30 (19)
C10—C11—C12—C13	−177.54 (19)	O10—N14—C46—C47	21.6 (3)
C19—N4—C13—N5	−0.7 (2)	O9—N14—C46—C45	24.8 (3)
Ni—N4—C13—N5	−163.04 (13)	O10—N14—C46—C45	−157.34 (19)
C19—N4—C13—C12	175.34 (16)	C45—C46—C47—C48	−0.8 (3)
Ni—N4—C13—C12	13.0 (2)	N14—C46—C47—C48	−179.66 (18)
C14—N5—C13—N4	0.9 (2)	C46—C47—C48—C49	−2.5 (3)
C14—N5—C13—C12	−174.71 (18)	C46—C47—C48—N15	177.57 (19)
N3—C12—C13—N4	−17.1 (2)	O11—N15—C48—C49	−167.0 (2)
C11—C12—C13—N4	161.46 (19)	O12—N15—C48—C49	13.0 (3)
N3—C12—C13—N5	158.31 (19)	O11—N15—C48—C47	13.0 (3)
C11—C12—C13—N5	−23.2 (3)	O12—N15—C48—C47	−167.0 (2)
C13—N5—C14—C15	178.0 (2)	C47—C48—C49—C50	0.9 (3)
C13—N5—C14—C19	−0.7 (2)	N15—C48—C49—C50	−179.18 (19)
N5—C14—C15—C16	−179.7 (2)	C48—C49—C50—N16	−176.54 (18)
C19—C14—C15—C16	−1.2 (3)	C48—C49—C50—C45	4.2 (3)
C14—C15—C16—C17	−0.5 (3)	O14—N16—C50—C49	151.78 (19)
C15—C16—C17—C18	1.2 (3)	O13—N16—C50—C49	−26.2 (3)
C16—C17—C18—C19	−0.2 (3)	O14—N16—C50—C45	−28.9 (3)
C13—N4—C19—C18	179.4 (2)	O13—N16—C50—C45	153.13 (19)
Ni—N4—C19—C18	−26.0 (3)	O8—C45—C50—C49	170.2 (2)
C13—N4—C19—C14	0.2 (2)	C46—C45—C50—C49	−6.8 (3)
Ni—N4—C19—C14	154.82 (15)	O8—C45—C50—N16	−9.1 (3)
C17—C18—C19—N4	179.49 (19)	C46—C45—C50—N16	173.97 (17)
C17—C18—C19—C14	−1.4 (3)	C54—N18—C56—O16	−2.3 (4)
N5—C14—C19—N4	0.3 (2)	C55—N18—C56—O16	−178.0 (2)
C15—C14—C19—N4	−178.50 (18)	C51—N17—C53—O15	1.4 (7)
N5—C14—C19—C18	−178.95 (17)	C52—N17—C53—O15	176.0 (4)
C15—C14—C19—C18	2.2 (3)	C52'—N17'—C53'—O15'	1(2)
C26—N6—C20—C21	−178.0 (2)	C51'—N17'—C53'—O15'	−177.2 (11)

### Hydrogen-bond geometry (Å, °)

**Table:**

*D*—H···*A*	*D*—H	H···*A*	*D*···*A*	*D*—H···*A*
N7—H7A···O8	0.88	1.93	2.769 (2)	158
C24—H24A···O14	0.95	2.60	3.226 (3)	124
C28—H28A···O8	0.95	2.26	3.144 (2)	155
C51—H51A···O15	0.98	2.41	2.812 (4)	104
C51—H51C···O7	0.98	2.47	3.304 (4)	143
C54—H54A···O16	0.98	2.43	2.800 (3)	102
N2—H2B···O16^i^	0.88	1.91	2.777 (2)	170
C9—H9A···O16^i^	0.95	2.55	3.411 (3)	150
N5—H5B···O1^ii^	0.88	1.81	2.684 (2)	175
N10—H10B···O15^iii^	0.88	1.92	2.803 (3)	180
C10—H10A···O6^iv^	0.95	2.59	3.398 (3)	143
C55—H55A···O10^v^	0.98	2.49	3.326 (3)	143

Symmetry codes: (i) *x*−1/2, −*y*+3/2, *z*−1/2; (ii) *x*+1/2, −*y*+3/2, *z*−1/2; (iii) *x*+1/2, −*y*+3/2, *z*−3/2; (iv) −*x*+3/2, *y*+1/2, −*z*+3/2; (v) *x*, *y*, *z*+1.
